# Toscana virus meningo-encephalitis: an important differential diagnosis for elderly travellers returning from Mediterranean countries

**DOI:** 10.1186/s12877-017-0593-2

**Published:** 2017-08-29

**Authors:** James Veater, Farhan Mehedi, Chee Kay Cheung, Laura Nabarro, Jane Osborne, Nicholas Wong, Martin Wiselka, Julian W Tang

**Affiliations:** 1 Infectious Disease Unit, University Hospitals of Leicester, NHS Trust, Leicester, UK; 2 Diabetes and Endocrinology, University Hospitals of Leicester, NHS Trust, Leicester, UK; 3 John Walls Renal Unit, University Hospitals of Leicester NHS Trust, Leicester, UK; 4 Rare and Imported Pathogens Laboratory, Porton Down, Wiltshire, UK; 5 Clinical Microbiology, University Hospitals of Leicester, NHS Trust, Level 5 Sandringham Building, Leicester Royal Infirmary, Infirmary Square, Leicester, LE1 5WW UK; 6 Infection, Immunity and Inflammation, University of Leicester, Leicester, UK

**Keywords:** Case report, Toscana virus, Encephalitis, Meningitis, Sandfly, Returning traveller, Spain

## Abstract

**Background:**

Elderly patients have a long list of differentials for causes of acute confusion and altered consciousness levels, including infectious agents. In addition, elderly, retired patients often have more time to travel for tourism, particularly to exotic, warmer locations. Mediterranean countries such as Spain and Italy are popular holiday destinations for British and other tourists, especially during the winter months. However, these warm climates allow insect vectors to proliferate, increasing the risk of exposure to endemic vectorborne viral infections whilst on vacation. Such infections may not be routinely considered by geriatric medical teams.

**Case presentation:**

An 87-year old gentleman presented with a three-day history of worsening confusion, lethargy, ataxia, and fevers following a trip to Spain, where he may have sustained a sandfly bite. By the time of admission, he had a reduced GCS, was hallucinating, and was incontinent of urine and faeces, though blood pressure and heart rate were normal. He also appeared hyperaesthetic, and found even capillary blood sugar testing extremely painful. He had no history of cognitive defect or other neurological conditions. He had been previously independently active, with frequent trips to Spain where he maintained a holiday home. He probably sustained a sandfly bite during this most recent trip, whilst cleaning out a shed.

Acute and convalescent sera demonstrated IgG antibodies to Toscana virus at extremely high titres of ≥1:10,000 by immunofluorescence assay, though no Toscana virus RNA was detectable in these sera by the time of presentation.

**Conclusions:**

Toscana virus should be included in the differential diagnosis of any patients presenting with meningo-encephalitis who have recently returned from a Mediterranean country. Testing for Toscana virus infection is performed by serological testing on acute/convalescent paired sera, and/or a polymerase chain reaction (PCR) assay on blood or cerebrospinal fluid (CSF) if presenting within 5 days of illness onset. Making a diagnosis of Toscana virus meningitis/encephalitis (where no other pathogen is detected) has additional clinical utility in reducing or preventing unnecessary use of antibiotics, as well as reassuring the patient and family that generally, this illness is generally self-limiting and full recovery within a few weeks is expected, as in the case reported here.

## Background

An elderly patient with acute confusional state is a common presenting problem for geriatric medical teams with a wide range of causes, including central nervous system infections. The management of such patients can be problematic as investigations can be difficult to perform, particularly if they are invasive. Therefore, empirical antimicrobial therapy is often given without a microbiological diagnosis, making the choice and duration of therapy difficult to decide. In addition, as more elderly patients are maintaining independence and travelling abroad, imported infections can present to geriatric teams.

Here we describe a case where a non-invasive, serological test gave the diagnosis when invasive tests were not possible. We also highlight the need to obtain a travel history in this patient group. CARE guidelines were followed in the writing of this case report.

## Case presentation

An 87-year old gentleman presented to the geriatric medical team in June 2016 with a three-day history of worsening confusion, lethargy, ataxia, and fever (up to 38 °C) following a two-week trip to southern Spain.

By the time of admission, he had a GCS of 12 (M = 5, V = 4, E = 3), was hallucinating, and was incontinent of urine and faeces, though blood pressure and heart rate were normal. He also appeared hyperaesthetic, and seemed to find even capillary blood sugar testing extremely painful. He had no history of cognitive defect or other neurological conditions. He had been previously independently active, with frequent trips to Spain where he maintained a holiday home. He had cleaned out a shed during his last trip and we hypothesise he was likely to have sustained a sandfly bite during this.

His initial investigations showed a normal C-reactive protein (<5 mg/L) and white cell count (5.1 × 10^9^/L), with normal neutrophil (3.73 × 10^9^/L) and lymphocyte (1.07 × 10^9^/L) counts, but a mild thrombocytopenia (134 × 10^9^/L). A lumbar puncture was attempted but was unsuccessful, so no CSF examination was possible. However, an EEG showed no abnormal focal or epileptiform activity. A brain MRI showed age-related changes only. Malaria screen, urine and blood cultures, meningococcal and pneumococcal polymerase chain reaction (PCR) test results were all negative, as was a screen for human immunodeficiency virus.

He was treated with intravenous ceftriaxone (2 g, 12-hourly), amoxicillin (2 g, 4-hourly) and acyclovir (800 mg, 8-hourly) for 2 weeks and made a rapid improvement and returned to his normal state over a week, after which he was discharged home.

Acute and convalescent sera, taken 2 weeks apart, were sent to the Rare and Imported Pathogens Laboratory (Porton Down, Wiltshire, UK) for serological testing. IgG antibodies to Toscana and Naples virus were detected at titres of ≥1:10,000 (positive cut-off is 1:100 for this assay) by immunofluorescence assay on both samples (Euroimmun, Lübeck, Germany**)**. No other vectorborne pathogen was found in the routine serological panel testing.

We were unable to demonstrate the presence of IgM as, at the time of writing, there are no reliable assays for this. Toscana virus RNA, tested by an in-house Toscana-specific PCR, was undetectable on the initial sample. However this was taken 5 days into illness when the viral RNA is often no longer detectable.

We acknowledge we were unable to demonstrate a rise in IgG or the presence of viral RNA to confirm the diagnosis, and we recognise there is serological cross-reactivity between Toscana and Naples viruses, including some local strains such as Grenada virus [1]. However, as only Toscana virus causes a neuroinvasive syndrome, and the IgG titre on the acute sample was extremely high (at least 100 times higher than the positive cut-off) it is highly unlikely that this was a non-specific reaction and very probable that this was an acute infection.

## Discussion and conclusions

Toscana virus is a *Phlebovirus* (family *Bunyaviridae*), transmitted via sandfly (*Phelobotomus spp)* bites, and is endemic to countries bordering the Mediterranean Sea (Portugal, Spain, France, Greece, Croatia, Cyprus, Turkey), where there is a high seropositivity rate. Infection rates are highest in summer when the sandfly is most active, making this virus among the top three causes of meningitis in this region during this season [2–4].

The incubation period is up to 2 weeks, with typical symptoms of meningo-encephalitis: headache, fever, neck rigidity, positive Kernig’s sign, tremors, reduced Glasgow Coma Scale score, paresis and nystagmus [2]. Hyperaesthesia has been described and was seen in our patient [5]. Symptoms usually resolve over 7 days with no long term sequelae, though changes in personality after Toscana encephalitis have been reported [6]. There is currently no specific treatment or vaccine.

Diagnosis can be made by demonstrating a rise in Toscana virus IgG on serum, though there is cross-reactivity with other phleboviruses. Toscana virus can be identified using real-time PCR on CSF and serum only during acute infection, as the viraemia is short-lived. Only a few cases of Toscana infection are diagnosed each year in the UK and it is not a routine consideration in the differential diagnosis of viral meningo-encephalitis. Although a recent case has been reported in a traveller returning to the UK from Italy [7], this appears to be the first published case of Toscana virus in a traveller returning to the UK from Spain.

The purpose of this case report is to increase the awareness of Toscana virus infection, and the need to consider imported infection in the differential diagnosis of febrile elderly patients with confusion, particularly in elderly travellers who may travel to exotic warmer locations during their retirement. Making a diagnosis of Toscana virus meningitis/encephalitis (where no other pathogen is detected) can have a useful clinical impact by reducing or preventing unnecessary use of antibiotics, as well as reassuring the patient and family that generally, this illness is self-limiting with a full recovery expected within a few weeks, as in the case reported here.

## Abbreviations

CSF: Cerebrospinal fluid
PCR: Polymerase chain reaction
RNA: Ribonucleic acid
UK: United Kingdom
